# Age-related differences in strategic competition

**DOI:** 10.1038/s41598-021-94626-2

**Published:** 2021-07-28

**Authors:** Sebastian S. Horn, Judith Avrahami, Yaakov Kareev, Ralph Hertwig

**Affiliations:** 1 Department of Psychology, Developmental Psychology: Adulthood, University of Zurich, Binzmuehlestr. 14 (Box 11), 8050 Zurich, Switzerland; 2 Center for Adaptive Rationality, Max Planck Institute for Human Development, Berlin, Germany; 3 Federmann Center for the Study of Rationality, The Hebrew University of Jerusalem, Jerusalem, Israel

**Keywords:** Psychology and behaviour, Human behaviour, Cognitive ageing

## Abstract

Understanding how people of different ages decide in competition is a question of theoretical and practical importance. Using an experimental laboratory approach, this research investigates the ability of younger and older adults to think and act strategically with equal or unequal resources. In zero-sum games of resource allocation, younger adults (19–35 years) and older adults (65–81 years) made strategic decisions in competition against opponents of a similar age (Study 1; *N* = 120) or different age (Study 2; *N* = 120). The findings highlight people’s ability to make good interpersonal decisions in complex scenarios: Both younger and older adults were aware of their relative strength (in terms of material resources) and allocated their resources adaptively. When competing against opponents of a similar age, people’s gains were in line with game-theoretic predictions. However, younger adults made superior strategic allocations and won more frequently when competing against older adults. Measures of fluid cognitive and numerical abilities correlated with strategic behavior in interpersonal competition.

Much political and economic power is concentrated in the hands of older adults: with a median age of 63 years, influential leaders and businesspeople are older than most citizens in their countries, according to a Forbes ranking of the “world’s most powerful people”^1^. Due to demographic transitions in many industrialized countries, the proportion of older adults who make strategic political, financial, and other decisions is set to increase even further^2^. An important class of strategic decisions involves competition, in which the competing parties can invest limited and different amounts of resources to achieve their goals (i.e., David-Goliath scenarios). That is, humans and other animals compete for survival, prosperity, or standing—but the means available for competition may differ dramatically. It is therefore a pertinent question whether and how people’s strategic allocation decisions differ as a function of age and with the material resources they hold. Yet surprisingly little research has addressed how individual differences (e.g., declining cognitive abilities in old age) may impact the outcome of competitions. In this article, we compare strategic allocation decisions by people of different ages when endowed with the same or different amounts of material resources.

The *preference* to compete (competitiveness) appears to differ with age. Mayr et al.^3^, for instance, found that competitiveness follows an inverted U-shaped pattern across adulthood with a peak in the fifties. However, little is known about age differences in *strategic performance* in competitions, in which a decision maker and other agents are mutually affected by their choices (cf.^4–9^). The main goal of our research was therefore to investigate younger and older adults’ interpersonal decisions in a game-theoretically formalized competition that requires strategic allocations.

## Strategic resource allocation

We study strategic allocations in competition with the Colonel Blotto game^10^. In this classic two-person zero-sum game, players simultaneously distribute their material resources (“troops”) across a set of fields (“battlefields”); battles are then waged over all or a subset of the fields. The game models many real-world situations in which competitors face the challenge of allocating their limited resources wisely to maximize their chances of success across different dimensions relevant to a competition (e.g., distributing funds in political campaigns; advertising across localities; economic investments). The Colonel Blotto game has been studied both theoretically^11–14^ and experimentally^15–17^. We investigated a variant of the game in which two players privately allocated their resources across a fixed number of fields. The players were endowed with either equal or unequal resources at the outset. Competition was then resolved over one randomly selected field and the player who allocated more resources on that field won the round. To preview the gist of game-theoretic reasoning^12,18^, players endowed with more resources (stronger players) should allocate resources following a uniform distribution on each field on which competition may occur. Players endowed with fewer resources (weaker players) should strategically concentrate their resources on fewer fields, abandoning some fields altogether in order to effectively compete with stronger players on the remaining fields (see section Game-Theoretic Benchmarks for details).

## Aging and strategic competition

How does aging affect competition that requires strategic interaction? We investigated three hypotheses. First, according to the *declining-strategic-cognition hypothesis*, performance in complex competitive games requires fluid cognitive and numerical abilities, which decline with age (e.g.,^19–21^). In line with this, prior findings indicate that differences in decision quality are often mediated by fluid cognitive abilities—particularly when tasks are complex and demanding^22^. The decline-in-strategic-cognition hypothesis would predict that older adults have more difficulties than younger adults to apply effective strategies in the competition game and play less systematically. Specifically, older adults may have more difficulties than younger adults to adaptively concentrate their resources on fewer fields as the weaker players and to cover all fields as the stronger players.

A second hypothesis suggests that younger and older adults differ in strategic allocations not because of their cognitive abilities—but because preferences may change with age. In many decision contexts, older adults tend to focus on accuracy and on preventing errors (e.g.,^23^), suggesting that aging gives rise to a cautious mindset. Moreover, aging has been associated with a decline in self-reported risk taking^24^. In the Colonel Blotto game, a lower propensity to take risks would mean that people avoid losing on fields by leaving them empty, but instead follow a risk-diversification strategy. The *increasing-cautiousness hypothesis* therefore suggests that older adults generally allocate resources more evenly than younger adults. The first two hypotheses predict age-related decline in strategic performance—but postulate different underlying mechanisms.

A third hypothesis suggests that older adults perform as well as (or possibly better) than younger adults in the Colonel Blotto game because they may be able to co-opt strategies that have proven valuable for dealing with limited and declining resources in general. Prior research indicates that lifelong experience helps older adults to approach problems pragmatically and wisely (e.g.,^25^). As people age, experiences of limitations and loss become increasingly pervasive in various domains of life^26^. It has been suggested that successful aging involves strategies of life management, including selectivity, which help older adults to deal with losses and allocate remaining resources efficiently^27^. As resources become more constrained, strategic selection can help older adults to reduce the number of options across which their resources are spread. The *successful-selection hypothesis* therefore predicts that older adults adopt a less-is-more strategy in the Colonel Blotto game, particularly as the weaker players. That is, older adults may selectively invest their limited resources across fewer fields and subsequently perform as well as (or possibly even better than) younger adults.

We examined these hypotheses in two studies in which participants competed for real money, based on their strategic allocations. In both studies, participants were randomly assigned to compete against opponents whose resources were unequal (*asymmetric condition*) or equal (*symmetric condition*) to their own. In Study 1, younger and older adults played multiple rounds of the Colonel Blotto game against opponents of a similar age. In Study 2, younger adults played the same version of the game against older adults.

## Study 1

Study 1 addressed the following research questions: Do people make systematic allocations in the Colonel Blotto game? How tuned are younger and older players to their strength relative to that of their opponents? How frequently do weaker players win in asymmetric competition? Are those wins in line with game-theoretic expectations? Finally, we examined the relation between cognitive abilities and strategic performance.

### Method

#### Participants

Participants in Study 1 were 60 younger and 60 older community-dwelling adults. In Study 1, each session involved homogeneous age groups of either younger or older adults. Participant characteristics and measures of fluid cognitive abilities, knowledge, affective state, numeracy, risk taking, and social value orientation are reported in Tables 1 and 2. The Supplemental Online Materials include further information about the test scales used in the current studies. All participants were paid volunteers, provided informed consent, and were recruited through local advertisements or from a database maintained by the research institute.

**Table 1:** Sociodemographic characteristics of participants in studies 1 and 2.

	Younger adults	Older adults
**Study 1**		
*N*	60	60
Age in years (mean/range)	26.6/19–35	70.4/65–80
Sex (female/male)	34/26	29/31
Education: Completed …		
Elementary school	5	23
High school	20	7
University/college	35	30
Wealth		
< €5000	24	10
€5000–€10,000	10	3
€10,000–€100,000	22	24
> €100,000	3	20
**Study 2**		
*N*	60	60
Age in years (mean/range)	24.1/19–31	71.3/67–81
Sex (female/male)	31/29	31/29
Education: completed …		
Elementary school	5	19
High school	35	4
University/college	20	37
Wealth		
< €5000	26	13
€5000–€10,000	9	4
€10,000–€100,000	11	21
> €100,000	11	17

Wealth = estimated overall current assets minus overall debts in € (95% of participants provided information about wealth).

**Table 2:** Test scales scores and actual wins for younger adults, older adults, and age-group comparisons.

	Younger adults		Older adults		Age-group comparison		
	*M*	*SD*	*M*	*SD*	Effect size *d*	95% CI	*p*
**Study 1**							
Test scales							
Crystallized abilities (0–37)	31.13	3.01	33.30	2.35	− 0.80	[− 1.17, − 0.43]	< 0.001
Fluid abilities: speed (0–133)	86.55	15.38	57.68	12.86	2.04	[+ 1.59, + 2.48]	< 0.001
Fluid abilities: reasoning (0–15)	11.82	1.81	7.70	2.91	1.70	[+ 1.27, + 2.13]	< 0.001
Numeracy (0–11)	9.52	1.32	7.88	2.79	0.75	[+ 0.37, + 1.12]	< 0.001
Positive affect: before game (1–5)	2.99	0.59	3.18	0.64	− 0.30	[− 0.66, + 0.06]	0.101
Negative affect: before game (1–5)	1.30	0.43	1.18	0.24	0.36	[− 0.00, + 0.72]	0.052
Positive affect: after game (1–5)	3.02	0.72	3.35	0.87	− 0.42	[− 0.78, − 0.05]	0.025
Negative affect: after game (1–5)	1.19	0.26	1.07	0.14	0.55	[+ 0.19, + 0.92]	0.003
Risk taking (0–10)	4.95	1.89	5.80	2.23	− 0.41	[− 0.77, − 0.05]	0.027
Social value orientation	28.35	11.34	21.02	15.66	0.54	[+ 0.16, + 0.91]	0.005
Actual wins							
48 vs. 48 (symmetric)	0.50	0.11	0.50	0.12	–	–	–
96 vs. 96 (symmetric)	0.50	0.08	0.50	0.10	–	–	–
48 vs. 96 (asymmetric, weaker)	0.27	0.08	0.25	0.08	0.25	[− 0.19, + 0.69]	0.260
96 vs. 48 (asymmetric, stronger)	0.73	0.08	0.75	0.09	− 0.24	[− 0.68, + 0.20]	0.282
**Study 2**							
Test scales							
Crystallized abilities (0–37)	30.77	2.59	33.45	1.88	− 1.19	[− 1.58, − 0.79]	< 0.001
Fluid abilities: speed (0–133)	86.17	16.38	60.68	13.07	1.72	[+ 1.29, + 2.14]	< 0.001
Fluid abilities: reasoning (0–15)	11.52	1.95	7.88	2.13	1.78	[+ 1.35, + 2.20]	< 0.001
Numeracy (0–11)	9.50	1.51	7.43	2.79	0.92	[+ 0.54, + 1.30]	< 0.001
Positive affect: before game (1–5)	2.95	0.56	3.24	0.61	− 0.50	[− 0.86, − 0.13]	0.007
Negative affect: before game (1–5)	1.32	0.33	1.18	0.23	0.50	[+ 0.14, + 0.86]	0.007
Positive affect: after game (1–5)	2.97	0.68	3.35	0.71	− 0.55	[− 0.91, − 0.18]	0.003
Negative affect: after game (1–5)	1.25	0.34	1.19	0.31	0.19	[− 0.17, + 0.54]	0.314
Risk taking (0–10)	4.88	1.66	5.84	2.15	− 0.50	[− 0.87, − 0.12]	0.009
Social value orientation	25.00	13.74	21.88	16.04	0.21	[− 0.16, + 0.57]	0.263
Actual wins							
48 vs. 48 (symmetric)	0.56	0.11	0.44	0.11	1.08	[+ 0.41, + 1.74]	0.002
96 vs. 96 (symmetric)	0.52	0.11	0.48	0.11	0.34	[− 0.29, + 0.96]	0.291
48 vs. 96 (asymmetric, weaker)	0.26	0.07	0.22	0.08	0.55	[+ 0.10, + 1.00]	0.016
96 vs. 48 (asymmetric, stronger)	0.78	0.08	0.74	0.10	0.45	[+ 0.002, 0.89]	0.049

Actual wins = observed proportion of wins out of 25 rounds. Details of the test scales used in both studies are in the Supplemental Online Materials.

All experiments and procedures were approved by an ethics committee at the Max-Planck-Institute for Human Development Berlin and were performed in accordance with the relevant guidelines and regulations.

#### Design

The game was introduced as a competition involving the allocation of points across several fields. The number of points a player could allocate in any given round (a player’s “strength”) changed from 48 to 96 points (or vice versa) halfway through the game; strength was manipulated within participants and counterbalanced (tests of the impact of order of available points in the game, i.e., 96–48 vs. 48–96 points, are in Supplemental Materials and did not indicate any significant main effects or interactions with other variables of interest). In each session participants were either of equal strength (symmetric control groups) or of unequal strength (asymmetric groups). In the asymmetric groups, half of the players were randomly assigned to be the stronger player (96 points) and the other half to be the weaker player (48 points). In order to have the same number of players facing opponents of equal strength, greater strength, and lesser strength in any between-subject comparison, the number of asymmetric groups was twice that of the number of symmetric groups (i.e., *n* = 80 players in asymmetric competition and *n* = 40 in symmetric competition). Including symmetric groups allowed us to disentangle the potential effects of the absolute number of points available from effects of the difference in points (relative strength) between the two opponents. Study 1 thus involved a 2 × 2 × 2 mixed design with *age group* (younger or older adults) and *opponent strength* (symmetric or asymmetric groups) as between-subject factors and *available resources* (48 or 96 points) as within-subjects factor.

#### Procedures and materials

**Figure 1:**
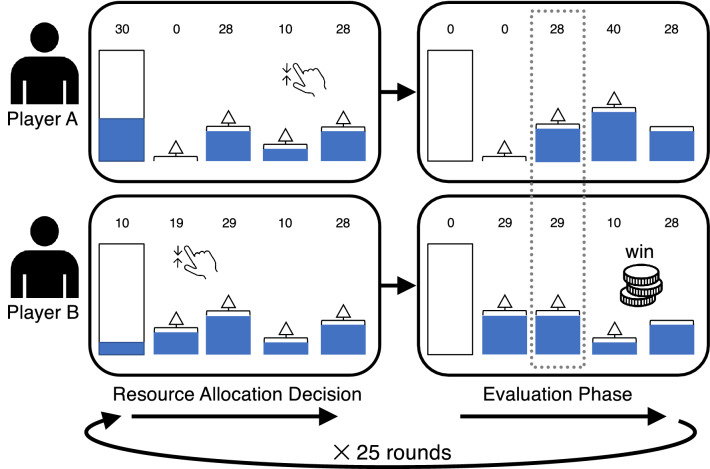
Schematic illustration of a sample trial from the allocation game (Colonel Blotto game). Verbatim instructions and details of procedures are provided in the Supplemental Materials. Participants allocated all of their resources (units) available in each round (depicted in a jar on the left side of the computer screen) across four fields (bins) as they saw fit, by moving corresponding sliders. After their allocation decision, participants clicked on a “next” button. In the evaluation phase, one of the four fields was then randomly selected by the program for comparison between two opponents. A player’s success was defined by a simple deterministic auction function. In the example, Player B wins the round (worth €0.2) against Player A, because Player B allocated more resources to that field than Player A did (29 vs. 28 units).

Groups of four players per session were seated in separate booths in the same laboratory room. Participants first read instruction slides on the computer screen describing the procedures and the structure of the allocation game. They were informed that they would compete against the other participants through connected computers and that this would be the only way of social interaction during the game. A trained experimenter also explained the game rules. Participants then completed a few practice rounds that were not incentivized to familiarize themselves with the procedures and had the opportunity to ask questions. They then played an incentivized version of the game (see Fig. 1): Each round involved a competition between two opponents who had to fully allocate their available resources (shown in a jar on the left side of the screen) as they saw fit across four fields by adjusting four corresponding sliders and then clicking on a “next” button. There was no time restriction for these allocation decisions. Once both players had made their allocations, the program randomly selected one of the four fields with equal probability, and the points allocated to that field by each opponent were displayed and compared. The player with more points on that field won €0.20; in the case of a tie, both players received €0.10. The game was repeated for a total of 25 rounds (with random rematching of opponents), followed by a short break. Participants were then informed that their available points per round would now change from 48 to 96 points (or vice versa). The game then continued for another 25 rounds. Each participant could earn a performance-contingent bonus of up to €10, depending on the number of wins. Finally, participants completed a battery of tests and questionnaires. Verbatim instructions and details of procedures are provided in Supplemental Materials.

### Results

In all analyses, the alpha level was set to 0.050. Sample-size planning was based on power analysis, which showed that 120 participants per study were sufficient to detect interaction effects between age groups and experimental factors of size $\eta_{p}^{2}$ = 0.04 with a power of 0.96 (further details are in the Supplemental Materials). CIs for effect sizes (*d* and $\eta_{p}^{2}$) were obtained using noncentral *t* and *F* distributions. We did not exclude any participants or trials from the analyses. Before presenting the empirical results, we first consider the game-theoretic benchmarks of the Colonel Blotto game.

#### Game-theoretic benchmarks

Finding a good approach to maximize success in the Colonel Blotto game is challenging. Given *k* fields and *m*_1_ and *m*_2_ resources available to two competitors, respectively, (with *m*_1_ ≥ *m*_2_ > 0), the strategy of dividing *m*_1_ resources equally among all fields could be easily foiled by an opponent simply allocating $\frac{{m_{1} }}{k} + 1$ resources to as many fields as possible. Competitors should therefore avoid predictability and use mixed strategies instead, where players choose from beneficial distributions of strategies (i.e., no pure Nash equilibria exist for the game^12,18^). Game-theoretic analyses suggest that, to maximize wins, a player in a symmetric competition and a stronger player in an asymmetric competition should allocate resources following a uniform distribution from zero to twice that player’s average resources on all fields on which competition may occur: ${\mathcal{U}}\left\{ {0,{ }\frac{{2m_{1} }}{k}} \right\}$. In contrast, a weaker player should leave a proportion of $1 - \frac{{m_{2} }}{{m_{1} }}$ fields empty to retain a chance of winning against a stronger opponent and allocate resources on the remaining fields following the strategy recommended for stronger players^12^.

#### Did players’ allocations differ from random play?

**Figure 2:**
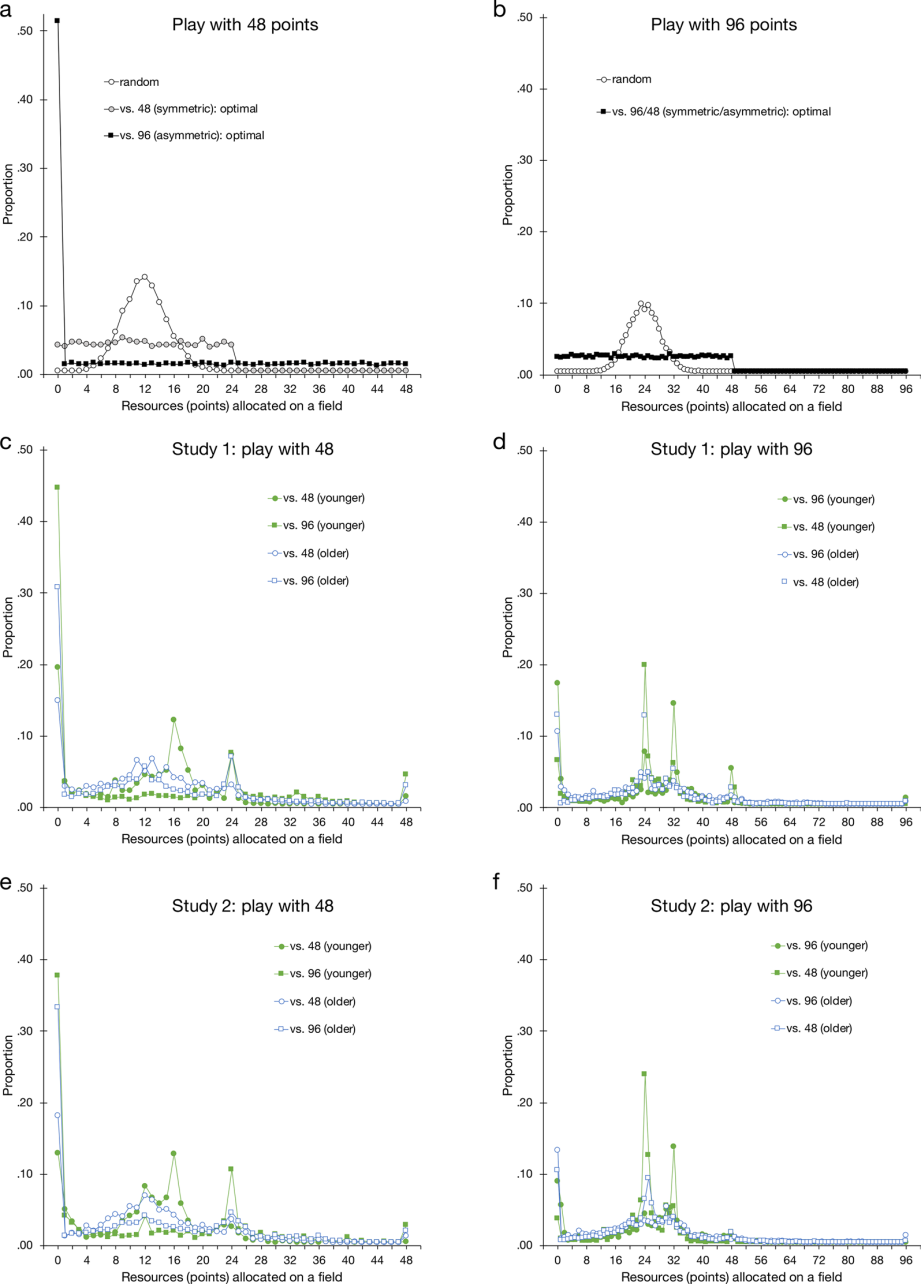
Proportions of allocations of a specific number of points on a field across the game for opponents of equal (symmetric) or unequal (asymmetric) strength, with either 48 or 96 points available. The upper two panels show simulated draws from game-theoretically optimal play and from random play with 48 points (panel **a**) or 96 points (panel **b**). The other panels plot the observed allocations of younger and older adults for Study 1 (homogeneous age groups; panels **c** and **d**) and Study 2 (mixed age groups; panels **e** and **f**) as a function of available points and opponent strength. For the simulations in panels **a** and **b**, we generated independent draws from distributions under optimal and random play (with *n* = 2000 and *n* = 4000 draws for the symmetric and asymmetric conditions, respectively, corresponding with the actual number of observations in each study). Random play implies that the opponents allocate their *m* available points unsystematically across *k* fields with equal probability, following a binomially distributed random variable $X \sim B\left( {m,{ }\frac{1}{k}} \right)$ for a given field.

In a Colonel Blotto game with *k* battlefields and *m* available points (resources), players can select from $$\left( {\begin{array}{*{20}c} {m + k - 1} \\ {k - 1} \\ \end{array} } \right)$$ strategies or allocation patterns^28^. In the present studies, for example, players with $m = 96$ available points have $$\left( {\begin{array}{*{20}c} {99} \\ 3 \\ \end{array} } \right) = 156,849$$ strategies at their disposal for allocating the points across $k = 4$ fields. This large strategy space makes it practically impossible for humans to consider and evaluate all possible strategies. One possibility is therefore that players distribute their points randomly across the fields due to the extreme complexity of the game. Random allocation might also be more pronounced in older than in younger adults (e.g., if decline in fluid cognitive abilities reduce strategically systematic play). Figure 2 shows random and game-theoretically optimal allocations (panels a–b), as well as participants’ actual allocations as a function of age, available points, and opponent strength (panels c–f). As can be seen, the actual allocations observed in both studies did not resemble the simulated random distributions. Instead, people distributed their points more uniformly and frequently allocated nothing to a field—a pattern that is very unlikely under random play. This finding suggests that neither younger nor older adults employed a random allocation strategy.

#### Allocation of resources

**Figure 3:**
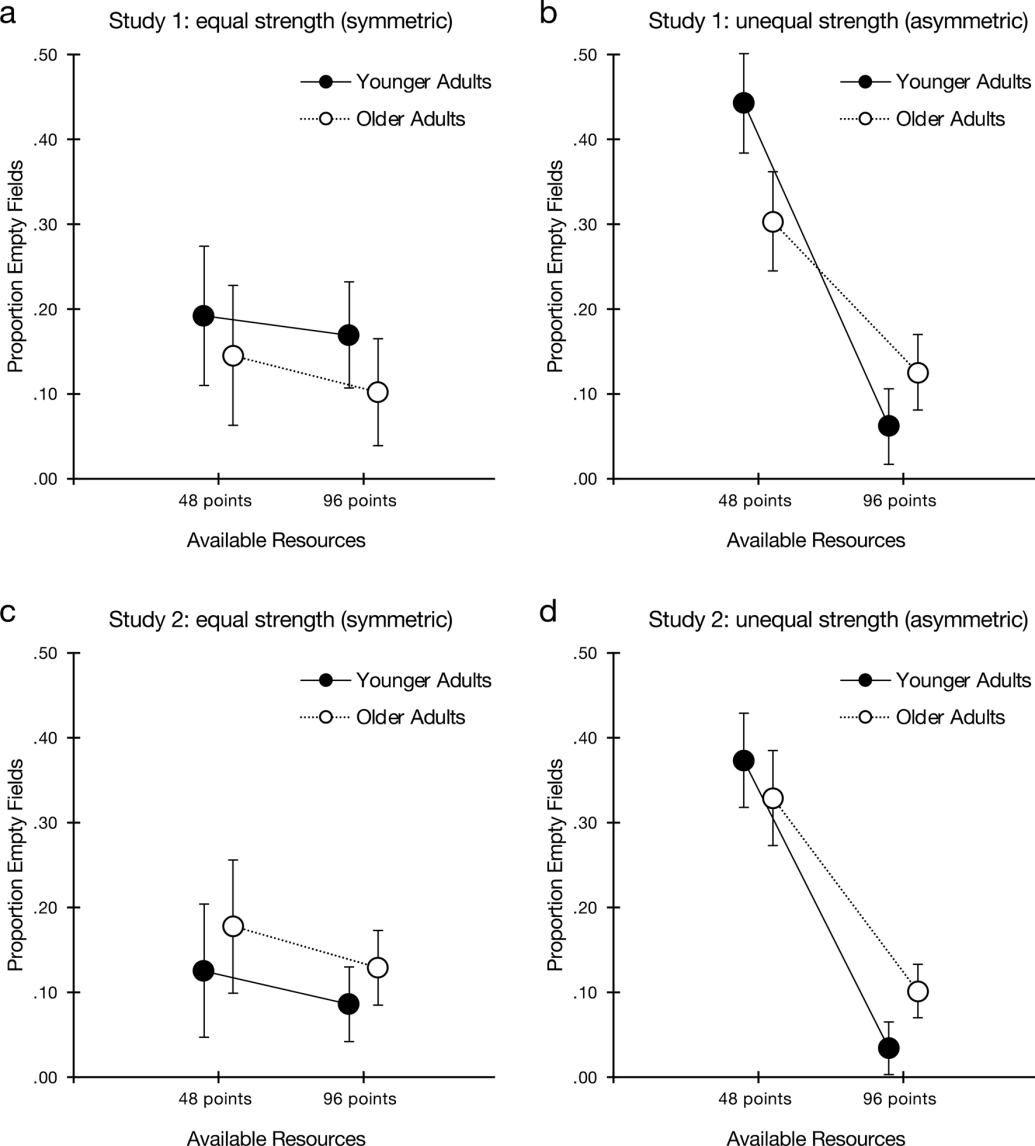
Strategic allocations (mean proportion of fields left empty) by younger and older adults in the Colonel Blotto game as a function of available resources in symmetric and asymmetric competition. Error bars represent 95% CIs.

The distributions of allocations in Fig. 2 already indicate that, in line with game-theoretic considerations^12^, both younger and older adults frequently left fields empty when they were in the role of the weaker player. For a more fine-grained quantitative analysis, we examined the proportion of fields left empty, which is a key indicator of strategic allocation behavior in this game^15,29^. Figure 3 presents the results for the two age groups in the symmetric and asymmetric conditions (panels a and b, respectively). Two findings are evident from Fig. 3 and were supported by a 2 (age group) × 2 (available resources) × 2 (opponent strength) factorial ANOVA: First, participants from both age groups were clearly sensitive to their relative strengths, leaving more fields empty when they had 48 points and were the weaker player than when they had 48 points and their strength was equal to that of their opponent. The interaction between available resources and opponent strength was significant, *F*(1, 116) = 60.34, *p* < 0.001, $\eta_{p}^{2}$ = 0.34, 95% CI = [0.21, 0.46]. Second, this effect was stronger among younger than older adults, as indicated by a three-way interaction between age group, available resources, and opponent strength, *F*(1, 116) = 12.40, *p* < 0.001, $\eta_{p}^{2}$ = 0.10, 95% CI = [0.02, 0.21]. Younger adults’ allocations thus corresponded better to the game-theoretic benchmark than older adults’ allocations did.

There was no significant main effect of age group, *F*(1, 116) = 2.93, *p* = 0.090, and no interaction between age group and opponent strength (*F* < 1), but there was a significant interaction between age group and available resources, *F*(1, 116) = 8.18, *p* = 0.005, $\eta_{p}^{2}$ = 0.07, 95% CI = [0.01, 0.17]. Unsurprisingly, the main effects of available resources, *F*(1, 116) = 96.98, *p* < 0.001, $\eta_{p}^{2}$ = 0.46, 95% CI = [0.32, 0.56], and of opponent strength, *F*(1, 116) = 8.56, *p* = 0.004, $\eta_{p}^{2}$ = 0.07, 95% CI = [0.01, 0.17] were both significant.

Follow-up tests indicated that in symmetric competition, the proportion of empty fields did not differ between younger and older adults, *F*(1, 38) = 1.30, *p* = 0.261. In asymmetric competition, when they were the weaker player, younger adults left more fields empty than older adults, *t*(75.51) = 3.15, *p* = 0.002, *d* = 0.70, 95% CI = [0.25, 1.15]; when they were the stronger player, younger adults left fewer fields empty than older adults, *t*(67.96) =  − 2.25, *p* = 0.028, *d* =  − 0.50, 95% CI = [− 0.95, − 0.05]. This pattern is consistent with the *decline-in-strategic-cognition hypothesis*.

In terms of game-theoretic benchmarks, when they were the weaker player in asymmetric competition, younger adults left slightly fewer than 50% fields empty, *t*(39) =  − 2.04, *p* = 0.049, *d* =  − 0.32, 95% CI = [− 0.64, − 0.002], and older adults left substantially fewer than 50% fields empty, *t*(39) =  − 5.79, *p* < 0.001, *d* =  − 0.92, 95% CI = [− 1.28, − 0.54].

In both studies, we also examined further measures of strategic allocation (the coefficient of variation). Moreover, we analyzed the fields that participants left empty with a generalized logistic mixed-modeling approach. The results of these additional analyses are reported in the Supplemental Materials and are in line with the findings reported here.

#### Achieved wins

When players have equal resources in symmetric competition in the zero-sum Colonel Blotto game, the average proportion of rounds won is by definition 0.50 (see ^29^). Therefore, in Study 1, it was only possible to examine age differences in wins for the asymmetric condition. Actual wins did not differ between younger and older adults (see Table 2). Regarding game-theoretically expected wins, when the players differ in strength, the weaker player should win half of the battles on fields not left empty^12^. Thus, weaker players (with 48 points) are expected to win 25% and stronger players (with 96 points) 75% of the rounds in the present study, if they follow game-theoretic prescriptions. Notably, people’s actual wins did not differ significantly from the expected wins in either age group in the asymmetric condition: all *t*s(39) < 1.36, all *p*s > 0.183. This result indicates that, regardless of age, people’s wins were in line with game-theoretic predictions.

#### Relation between wins and allocations

We further examined the relation between players’ wins and their allocations. As expected, the relation between rounds won and fields left empty depended on the type of competition (see Supplemental Materials for details): In asymmetric competitions, the relation between wins and fields left empty was moderately positive for weaker players, but negative for stronger players. In symmetric competitions, both relations were moderately negative. Thus, allocation strategies that covered all fields tended to yield beneficial outcomes for stronger players in both symmetric and asymmetric competition, but detrimental outcomes for weaker players in asymmetric competition.

#### Individual-difference measures

To what extent are strategy use and success in the competition game correlated with individual cognitive abilities, numeracy, risk preferences, and social value orientation? To address this question, we examined the intercorrelations between the fields left empty, achieved wins, and participants’ scores from psychometric tests (a) within the symmetric and asymmetric conditions and (b) within each age group, respectively (details are in Supplement 1). In the asymmetric conditions, numeracy and fluid cognitive abilities (cognitive speed and reasoning) correlated positively with wins in the game (by both stronger and weaker players) and with the fields left empty by weaker players—but negatively with the fields left empty by stronger players. Thus, participants who scored higher on these cognitive variables left more fields empty in the role of weaker players and covered more fields as stronger players (*r*s > 0.17, *p*s < 0.03). In the symmetric conditions, the patterns were less systematic (only the correlation between reasoning and wins with 48 points reached significance). When the correlations were examined separately within each age group, cognitive speed and numeracy correlated with older adults’ allocation behavior in asymmetric competition (the fields left empty by older adults as the weaker players; *r*s > 0.29, *p*s < 0.01), but in younger adults, these correlations failed to reach significance.

Overall, this suggests that fluid cognitive and numerical abilities are relevant predictors of strategic decisions, particularly in asymmetric competition. The correlational analyses are also in line with the declining-strategic-cognition hypothesis Strategic allocation behavior (within the age groups) tended to correlate with measures of fluid and numerical abilities; these measures, in turn, differed strongly between age groups (Table 2).

## Study 2

In Study 2, we investigated the role of opponent age. Rising through professional and social hierarchies takes time. The higher echelons of society thus tend to be populated by older people^1^, who interact strategically with people of a similar age, but also with people who are likely younger. Therefore, is strategic game play shaped by mismatch in players’ age? Three scenarios are conceivable: First, players of both age groups could play more strategically (allocate resources more competitively) against opponents from another age group than from their own age group. This pattern would be consistent with the idea that the similarity of an opponent (shared category membership) may attenuate the urge for competition^30,31^. Second, players of both age groups might generally play more strategically against younger than older adults, following shared norms of how one should interact with members of different age groups (e.g.,^32^) or stereotypical expectations that younger adults act more strategically than older adults^33^. This pattern would be evident in more strategic play of older adults and a deterioration in the play of younger adults relative to Study 1. Third, it is possible that age mismatch has no distinct influence on behavior in the game. Finally, the experimental setting in Study 2 allowed us to analyze the wins by different age groups also in symmetric competition, in contrast to Study 1 (where wins in symmetric competition were, by definition, 50% within each group).

### Method

#### Participants and procedures

In Study 2, 60 younger and 60 older adults (who did not take part in Study 1) participated for monetary compensation. Information about the sample is provided in Tables 1 and 2. The design, materials, and procedures were the same as in Study 1, with one important exception: Each session involved groups of two younger and two older adults. Participants were informed that they would compete against players of a different age group.

### Results

#### Allocation of resources

As shown Fig. 2, the allocation distributions in Study 2 (opponents of different age; panels e–f) were relatively similar to those in Study 1 (opponents of similar age; panels c–d), suggesting again that both younger and older adults were attuned to their opponent’s strength—but less so to their opponent’s age. Next, we report further analyses on the proportion of fields left empty and of wins in the game.

#### Proportion of fields left empty

Figure 3(panels c, d) shows that both age groups were attuned to their own and their opponent’s strength: The interaction effect between available resources and opponent strength was significant, *F*(1, 116) = 48.45, *p* < 0.001, $\eta_{p}^{2}$ = 0.30, 95% CI = [0.16, 0.41]. However, the three-way interaction between available resources, opponent strength, and age now failed to reach significance, *F*(1, 116) = 3.07, *p* = 0.083. There was no significant main effect of age group, *F*(1, 116) = 1.80, *p* = 0.183, and no interaction between age group and opponent strength (*F* < 1) or between age group and available resources *F*(1, 116) = 2.22, *p* = 0.139. Once again, the main effects of opponent strength, *F*(1, 116) = 13.19, *p* < 0.001, $\eta_{p}^{2}$ = 0.10, 95% CI = [0.02, 0.21], and available resources, *F*(1, 116) = 90.60, *p* < 0.001, $\eta_{p}^{2}$ = 0.44, 95% CI = [0.31, 0.54], were both significant.

Follow-up tests indicated that, in symmetric competition, the proportion of empty fields did not differ between younger and older adults, *F*(1, 38) = 1.60, *p* = 0.213. In asymmetric competition, when they were the weaker players, the age groups did not differ in the proportion of fields left empty, *t*(78) = 1.03, *p* = 0.305; in the role of the stronger player, younger adults left fewer fields empty than older adults, *t*(57.88) = 3.38, *p* = 0.001, *d* =  − 0.76, 95% CI = [− 1.21, − 0.29]. Both age groups left fewer than 50% of fields empty when they were the weaker player, *t*s(39) > 4.17, *p*s < 0.001, *d*s > 0.65.

#### Wins

Study 2 allowed for a direct comparison of wins when older adults played against younger adults in both symmetric and asymmetric competition: Younger adults achieved significantly more wins than older adults, except in symmetric competitions with 96 points (see Table 2).

Comparisons of game-theoretically expected and actual wins revealed that younger adults achieved more wins than expected in symmetric competition with 48 points, *t*(19) = 2.41, *p* = 0.026, *d* = 0.54, 95% CI = [0.06, 1.00], and in asymmetric competition with 96 points, *t*(39) = 2.25, *p* = 0.030, *d* = 0.35, 95% CI = [0.03, 0.67]. Correspondingly, older adults achieved fewer wins than expected with 48 points in symmetric and asymmetric competition.

#### The effect of opponent’s age

To examine if and how an opponent’s age affected strategic allocation decisions, we compared the proportion of fields left empty in Study 2 (age discordance) with Study 1 (age concordance; Supplement 4 provides details). The effect of opponent age was nonsignificant, *F*(1, 232) = 1.74, *p* = 0.189. However, there was an interaction between players’ age and opponent age, *F*(1,232) = 4.73, *p* = 0.031 , $\eta_{p}^{2}$ = 0.02, 95% CI = [0.00, 0.07]: Younger adults left fewer fields empty when playing against older opponents than when playing against opponents of the same age, *F*(1, 116) = 7.40, *p* = 0.008, $\eta_{p}^{2}$ = 0.06, 95% CI = [0.004, 0.16]. In contrast, older adults left as many fields empty in both situations (*F* < 1). There were no further interactions involving opponent age (all *F*s < 1.21; *p*s > 0.271). In sum, the analyses showed, first, that players did not act more strategically when playing against opponents of different age than they did when playing against opponents of similar age (the interaction between available resources, opponent strength, and opponent age was nonsignificant, *F* < 1). Second, players did not generally act more strategically against younger than older opponents (older adults’ strategic allocations were similar when playing against same-age and different-age opponents). Third, opponent age did affect strategic behavior in younger adults, who left fewer fields empty (and thus acted less strategically in asymmetric competition) when playing against older than younger opponents. Nonetheless, younger adults won more frequently than older adults in Study 2.

## General discussion

Competitors in the real world have limited and different amounts of resources available to achieve their goals (e.g.,^29,34^). For a good reason, research has linked strategic allocation of resources to success in many contexts, including economic, political, and military competition^11,13,14^. The current findings contribute to this research by investigating how aging and declining cognitive abilities may interfere with the ability to strategize when mustering one’s resources for a competitive situation. Our goal was to better understand age-related differences in strategic behavior and to examine the dynamics of competition when opponents hold different amounts of resources or are from different or similar age cohorts. We found that both younger and older adults acted strategically and left fields uncontested as the weaker opponents, quite closely following game-theoretic benchmarks. Younger adults nevertheless made strategically more successful allocations than older adults. Moreover, the strategic decisions of older adults were not affected by the age of their opponent; younger adults, in contrast, acted less strategically when they competed against older opponents.

These findings inform and integrate several lines of research. They inform research on the aging decision maker (e.g.,^22,23,35^) by demonstrating for the first time in the context of strategic interpersonal competition that both younger and older adults adaptively select strategies as a function of the resource environment they face (cf.^36^). This is not at odds with previous findings that older people may act more cautiously, are less prone to taking risks (e.g.,^24^), and exercise motivational selectivity (e.g.,^26,27,37,38^). Rather, in the game we studied, strategic cognition (in interaction with situational factors) took precedence over preferences such as risk aversion and cautiousness. Consistent with this, participants’ strategic allocations did not correlate with individual measures of value orientation^39^ or risk taking^40^ in the present studies. We note, however, that the relations among measures of risk taking and between chronological age and risk taking are generally complex. Correlations among different behavioral measures of risk-taking are usually small and task-specific variance is high^41^. Moreover, older adults typically indicate lower levels of risk taking than younger adults in self reports^24^, but the pattern is different in behavioral decision tasks^42,43^. Specifically, when risky decisions are made from description^44,45^ (as in the lotteries^40^ we used here to measure risk taking) there are no systematic age-related differences^42,46,47^. Taken together, the lack of correlations between strategic allocations in the game and risk taking is not at odds with prior research, but calls for a nuanced analysis of different aspects of risk taking in competitive environments.

Older players, like younger players, displayed the skill to act strategically. However, consistent with the declining-strategic-cognition hypothesis, we found age-related differences in strategic behavior along with moderate relations between older adults’ strategic behavior and their fluid cognitive and numerical abilities. This echoes research from other areas of decision making, which has shown that cognitive and numerical abilities account for age differences in the quality of decisions^19–21,48^. In the present studies, younger adults achieved more wins in competition and came closer to game-theoretic benchmarks than older adults. However, compared with the large effects of environmental factors (available resources and opponent strength) on competitive decisions, the role of cognitive decline was small. Specifically, we found that the environmental factors (and their interactions) together accounted for ca. 30% of the variance in people’s strategic allocations whereas chronological age (and interactions with age) accounted for ca. 3% of the variance. This highlights that younger and older adults are by and large competent competitors capable to adapt to circumstances at hand. It is possible that in competitions outside the laboratory, older adults may even retain a competitive edge through their accumulated knowledge or the skilled use of heuristic strategies (e.g.,^49,50^) that are well attuned to information-rich environments^36^. For example, experience (crystallized abilities) can help older adults to compensate for age-related decline in fluid abilities to make similarly good economic decisions as younger adults^51^. Thus, more work is needed to find out whether the age differences in strategic allocations observed here will generalize beyond the commonly abstract and context-free paradigms studied in experimental economics and game theory^52^. Whereas several studies^53–55^ suggest that strategic decisions in the laboratory generalize well to naturalistic field settings (for further discussion, see^56^), there is also evidence that the specific framing of a problem may strongly affect decision makers’ reasoning and strategies^57–59^. In a study with university students, different content descriptions in the Blotto game had no effect on wins, but on people’s allocations^15^. It remains to be seen how younger and older adults compete in differently framed domains, in which individual experience or propensities could play different roles.

Older players’ decisions were similar, regardless of their opponents’ age; younger players, in contrast, acted less strategically against older than same-age opponents. This suggests that shared category membership alone does not attenuate the sense of competition (e.g.,^31,32^). Rather, younger adults may operate according to certain norms (e.g., “don’t exploit older people”) or expectations (e.g., “older players won’t compete as fiercely”) pertaining to older adults (cf.^33,60^). Perhaps consistent with this possibility, we found that measures of other-regarding preferences and social value orientation^39^ tended to be higher in younger than in older adults (Table 2). Clearly, further research is needed to better understand the relation between measures of prosocial and competitive orientation^3^.

## Conclusion

Many important decisions are made by people who are relatively old. A better understanding of age differences in allocation decisions is important because they affect outcomes in economic and other competitions, in which resources are often asymmetrically distributed. Younger adults made strategically more successful allocations than older adults and strategic performance in older adults correlated with measures of fluid abilities. At the same time, our research demonstrates that younger and older players can decide, intuitively, in ways that approximate sophisticated game-theoretic solutions and highlights the ability of younger as well as older adults to reach good decisions—even in complex David-Goliath competitions.

## Supplementary Information

Supplementary Figures.
