# Supplementary material for: Age-related differences in strategic competition
Source: Sci Rep. 2021 Jul 28;11:15318. doi: 10.1038/s41598-021-94626-2 (PMC8319396; doi:10.1038/s41598-021-94626-2)
Supplement: Supplementary file 1 — Supplementary Figures. [file 41598_2021_94626_MOESM1_ESM.docx]

**Supplemental Materials
Age-related differences in strategic competition**

**Supplement 1: Correlational Analyses of**

**Strategic Behavior, Achieved Wins, and Individual Scores from Psychometric Tests**

**

**Figure S1.** Zero-order correlations in the asymmetric game conditions.

**Figure S2.** Zero-order correlations in the symmetric game conditions.

**Figure S3.** Zero-order correlations: Younger adults in the asymmetric game conditions.

**Figure S4.** Zero-order correlations: Older adults in the asymmetric game conditions.

**Figure S5.** Zero-order correlations: Younger adults in the symmetric game conditions.

**Figure S6.** Zero-order correlations: Older adults in the symmetric game conditions.

**Supplement 2: Description of Psychometric Test Scales**

Each participant in Study 1 and 2 completed a battery of standard psychometric tests measuring crystallized knowledge, fluid cognitive abilities, numeracy, positive and negative affect, risk attitude, and social value orientation.

**Crystallized Knowledge (Vocabulary)**

In a paper-and-pencil spot-a-word vocabulary test, participants completed 37 items of increasing difficulty. Each item consisted of one word and four nonwords. Participants were asked to select the word (and to guess if they did not know the correct answer). Vocabulary tests have been shown to correlate highly with other tests of crystallized intelligence and semantic knowledge (e.g., ^1,2^).

**Fluid Cognitive Abilities**

**Cognitive Speed.** We used a paper-and-pencil version of the Digit Symbol Substitution Test (adapted from WAIS-IV; cf. ^3^) as an indicator of cognitive speed. In this speed test, all participants completed as many items as possible within 2 min.

**Reasoning Matrices.** We used an adapted paper-and-pencil subscale of the Culture Fair Intelligence Test CFT-20 to measure abstract reasoning as an indicator for nonverbal fluid intelligence^4^. All participants first completed three practice items and then had 3 min to complete up to 15 items of increasing difficulty. Each item consisted of a 2 × 2 matrix or 3 × 3 matrix of visual geometric patterns with one missing pattern in the bottom right corner of the matrix. Participants completed the matrix by selecting one of five response options.

**Numeracy.** We used an 11-item scale^5^ to measure the ability to understand and use numeric information (numeracy). The scale includes questions in different formats (free responses and multiple-choice) to quantify the basic understanding of fractions, chance, proportions, and percentages (e.g., “If the chance of getting a disease is 20 out of 100, this would be the same as having a ___% chance of getting the disease”).

**Positive and Negative Affect**

We used the Positive and Negative Affect Scale (PANAS)^6^ to measure positive and negative affect before and after the game. Two 10-item subscales measure positive and negative affect, respectively. On each item, participants rated the degree to which a given adjective (e.g., “active,” “ashamed”) described their current state on a 5-point scale ranging from 1 (not at all) to 5 (very much). We used affect assessed before the game to predict game behavior in our correlational analyses.

**Risk Attitude**

Participants made 10 hypothetical choices between two monetary lotteries (see ^7^). The number of risky choices (out of 10 items) served as a behavioral measure of risk attitude (higher scores indicate a higher preference for risky options).

**Social Value Orientation**

Social value orientation (SVO) was measured by six primary items of the SVO slider measure^8^, which was developed to assess social goals in interdependent decisions. On each item, participants indicated how they would divide the available resources between themselves and another person. The SVO scale distinguishes four common social value orientations: altruistic, prosocial, individualistic, and competitive. SVO can be scored in a single index (SVO angle; for details, see Murphy et al., 2011). Altruists have an SVO angle greater than 57.15°; prosocials have angles between 22.45° and 57.15°; individualists have angles between –12.04° and 22.45°; and competitive types have an angle less than −12.04°. Adequate test–retest reliability (*r* = 0.91) and convergent validity with other measures of social value orientation has been established.

**Supplement 3: Analyses of Further Allocation Measures**

To assess the robustness of our main findings, we also examined further measures of players’ strategic allocation decisions in Study 1 and 2. Specifically, in addition to the proportion of fields left empty (*P*_empty_) in the Colonel Blotto game, we used the coefficient of variation (*CVAR*) to quantify the average variability of points allocated to a given field across the rounds. *CVAR* is a standardized measure of dispersion and is defined as the ratio of the standard deviation to the mean: $CVAR=\frac{SD}{M}$. Thus, *CVAR* takes into account both the variability in allocated points (which was higher when people left a field occasionally empty) and the average resources assigned to a field. The measures *P*_empty_ and *CVAR* were highly correlated (all *r*s > .76, all *p*s < .001). The supplementary analyses thus yielded similar results on older and younger adults’ allocation decisions to those reported in the main text, speaking to the robustness of the main findings.

**Table S3.** *Further Measures of Strategic Allocations in Study 1.*

| **Coefficient of Variation** | *SS* | *df* | *MS* | *F* | *p* | η_p_² |
| --- | --- | --- | --- | --- | --- | --- |
| Within-Subjects Effects |  |  |  |  |  |  |
| Resources | 6.91 | 1 | 6.91 | 121.90 | < .001 | .51 |
| Resources×OpponentStrength | 5.05 | 1 | 5.05 | 89.13 | < .001 | .44 |
| Resources×AgeGroup | 0.78 | 1 | 0.78 | 13.71 | < .001 | .11 |
| Resources×OpponentStrength×AgeGroup | 0.75 | 1 | 0.75 | 13.24 | < .001 | .10 |
| Residual | 6.57 | 116 | 0.06 |  |  |  |
| Between-Subjects Effects |  |  |  |  |  |  |
| OpponentStrength | 0.68 | 1 | 0.68 | 4.56 | .035 | .04 |
| AgeGroup | 0.17 | 1 | 0.17 | 1.15 | .287 | .01 |
| OpponentStrength×AgeGroup | 0.04 | 1 | 0.04 | 0.27 | .604 | .00 |
| Residual | 17.24 | 116 | 0.15 |  |  |  |

*Note.*  Type III sum of squares; *SS* = sum of squares; *MS* = mean square

**Table S4.** *Further Measures of Strategic Allocations in Study 2.*

| **Coefficient of Variation** | *SS* | *df* | *MS* | *F* | *p* | η_p_² |
| --- | --- | --- | --- | --- | --- | --- |
| Within-Subjects Effects |  |  |  |  |  |  |
| Resources | 7.49 | 1 | 7.49 | 136.38 | < .001 | .54 |
| Resources×OpponentStrength | 5.77 | 1 | 5.77 | 105.10 | < .001 | .48 |
| Resources×AgeGroup | 0.45 | 1 | 0.45 | 8.28 | .005 | .07 |
| Resources×OpponentStrength×AgeGroup | 0.54 | 1 | 0.54 | 9.90 | .002 | .08 |
| Residual | 6.37 | 116 | 0.06 |  |  |  |
| Between-Subjects Effects |  |  |  |  |  |  |
| OpponentStrength | 0.53 | 1 | 0.53 | 5.58 | .020 | .05 |
| AgeGroup | 0.33 | 1 | 0.33 | 3.45 | .066 | .03 |
| OpponentStrength×AgeGroup | 0.04 | 1 | 0.04 | 0.46 | .500 | .00 |
| Residual | 11.10 | 116 | 0.10 |  |  |  |

*Note.*  Type III sum of squares; *SS* = sum of squares; *MS* = mean square

**Supplement 4: Comparison Between Study 1 and 2**

To examine the effect of *Opponent Age* on competitive behavior, we analyzed the combined data from Study 1 and 2 and all *N* = 240 participants. We again examined two criterion variables of strategic behavior in the Colonel Blotto game: the proportion of fields left empty and the coefficient of variation (*CVAR*; measuring the average variability of points allocated to a given field across rounds). Overall, the pattern of strategic allocations across studies was relatively similar in both same-age and different-age scenarios. However, there was a significant interaction between *Opponent Age* and *Age Group*, indicating that younger adults tended to leave fewer fields empty when playing against older opponents than when playing against opponents of the same age group.

**Table S5.** *Measures of Strategic Allocations with Opponent Age as Additional Factor*

| **Proportion of Fields Left Empty** | *SS* | *df* | *MS* | *F* | *p* | η_p_² |
| --- | --- | --- | --- | --- | --- | --- |
| Within-Subjects Effects |  |  |  |  |  |  |
| Resources | 2.73 | 1 | 2.73 | 186.97 | < .001 | .45 |
| Resources×OpponentAge | 0.00 | 1 | 0.00 | 0.10 | .749 | .00 |
| Resources×OpponentStrength | 1.57 | 1 | 1.57 | 107.81 | < .001 | .32 |
| Resources×AgeGroup | 0.13 | 1 | 0.13 | 9.20 | .003 | .04 |
| Resources×OpponentAge×OpponentStrength | 0.00 | 1 | 0.00 | 0.02 | .881 | .00 |
| Resources×OpponentAge×AgeGroup | 0.01 | 1 | 0.01 | 0.71 | .400 | .00 |
| Resources×OpponentStrength×AgeGroup | 0.20 | 1 | 0.20 | 13.50 | < .001 | .06 |
| Resources×OpponentAge×OpponentStrength×AgeGroup | 0.02 | 1 | 0.02 | 1.21 | .272 | .01 |
| Residual | 3.39 | 232 | 0.02 |  |  |  |
| Between-Subjects Effects |  |  |  |  |  |  |
| OpponentAge | 0.06 | 1 | 0.06 | 1.74 | .189 | .01 |
| OpponentStrength | 0.69 | 1 | 0.69 | 20.70 | < .001 | .08 |
| AgeGroup | 0.01 | 1 | 0.01 | 0.26 | .614 | .00 |
| OpponentAge×OpponentStrength | 0.00 | 1 | 0.00 | 0.00 | .975 | .00 |
| OpponentAge×AgeGroup | 0.16 | 1 | 0.16 | 4.73 | .031 | .02 |
| OpponentStrength×AgeGroup | 0.00 | 1 | 0.00 | 0.06 | .813 | .00 |
| OpponentAge×OpponentStrength×AgeGroup | 0.02 | 1 | 0.02 | 0.61 | .435 | .00 |
| Residual | 7.73 | 232 | 0.03 |  |  |  |
| **Coefficient of Variation** |  |  |  |  |  |  |
| Within-Subjects Effects |  |  |  |  |  |  |
| Resources | 14.39 | 1 | 14.39 | 257.95 | < .001 | .53 |
| Resources×OpponentAge | 0.01 | 1 | 0.01 | 0.11 | .744 | .00 |
| Resources×OpponentStrength | 10.81 | 1 | 10.81 | 193.77 | < .001 | .46 |
| Resources×AgeGroup | 1.21 | 1 | 1.21 | 21.69 | < .001 | .09 |
| Resources×OpponentAge×OpponentStrength | 0.01 | 1 | 0.01 | 0.22 | .641 | .00 |
| Resources×OpponentAge×AgeGroup | 0.02 | 1 | 0.02 | 0.38 | .536 | .00 |
| Resources×OpponentStrength×AgeGroup | 1.29 | 1 | 1.29 | 23.04 | < .001 | .09 |
| Resources×OpponentAge×OpponentStrength×AgeGroup | 0.01 | 1 | 0.01 | 0.15 | .701 | .00 |
| Residual | 12.95 | 232 | 0.06 |  |  |  |
| Between-Subjects Effects |  |  |  |  |  |  |
| OpponentAge | 0.43 | 1 | 0.43 | 3.50 | .063 | .02 |
| OpponentStrength | 1.21 | 1 | 1.21 | 9.88 | .002 | .04 |
| AgeGroup | 0.01 | 1 | 0.01 | 0.11 | .743 | .00 |
| OpponentAge×OpponentStrength | 0.00 | 1 | 0.00 | 0.03 | .852 | .00 |
| OpponentAge×AgeGroup | 0.49 | 1 | 0.49 | 3.99 | .047 | .02 |
| OpponentStrength×AgeGroup | 0.08 | 1 | 0.08 | 0.69 | .408 | .00 |
| OpponentAge×OpponentStrength×AgeGroup | 0.00 | 1 | 0.00 | 0.00 | .985 | .00 |
| Residual | 28.34 | 232 | 0.12 |  |  |  |

*Note.*  Type III sum of squares; *SS* = sum of squares; *MS* = mean square

**Supplement 5: Sample Size Planning**

We conducted statistical power analysis to inform our sample-size planning for the current studies. Previous empirical studies with the Colonel Blotto game (cf. ^9,10^) have reported medium-to-large effect sizes. In the current study, we aimed at detecting smaller effect sizes than in this previous research (i.e., small-to-medium effects, following the classification by Cohen et al., 2003) of size $\eta_{p}^{2}$ = .04 (or *f* ~ .20) for interactions between *Age Group* and the experimental factors (*Resources* and *Opponent Strength*) with a statistical power of at least .90 (assuming α = .05). With a total sample size of *N* = 120 in each study, the achieved statistical power to detect such effect sizes was 1−β = .96. Figure S1 shows the power to detect interaction effects of different sizes in the ANOVA models as a function of sample size.

**Figure S7**. Statistical Power to detect Age × Treatment effects of different sizes as a function of total sample size for the ANOVA models in the current studies.

**Supplement 6: Zero-Order Correlations Between Allocations and Wins**

This supplement includes zero-order correlations between allocation (proportions of empty fields; coefficient of variation) and wins (actual and potential proportion of round wins). In both studies, we used the same procedures to collect the same measures from each participant. Assuming that relations between basic cognitive abilities and game behavior held across studies, we combined the data from both studies to obtain more stable estimates from the correlational analyses^11^.

In asymmetric competitions, the statistical relation between wins and empty fields tended to be positive for weaker players but negative for stronger players. In symmetric competitions, these relations tended to be moderately negative.

Because actual wins in the current version of the Colonel Blotto game are somewhat noisy (a player’s strategy is evaluated on just one of four possible fields in 25 rounds), we also considered potential wins as a possibly more stable estimate of the players’ strategic performance. To this end, we averaged the frequency of wins a player’s allocation would have yielded in a round, had it been compared against the opponent’s allocation on all four possible field pairs. Potential wins were significantly correlated with actual wins, and the analyses with the two measures led to the same conclusions. Therefore, we focus on actual wins in the article.


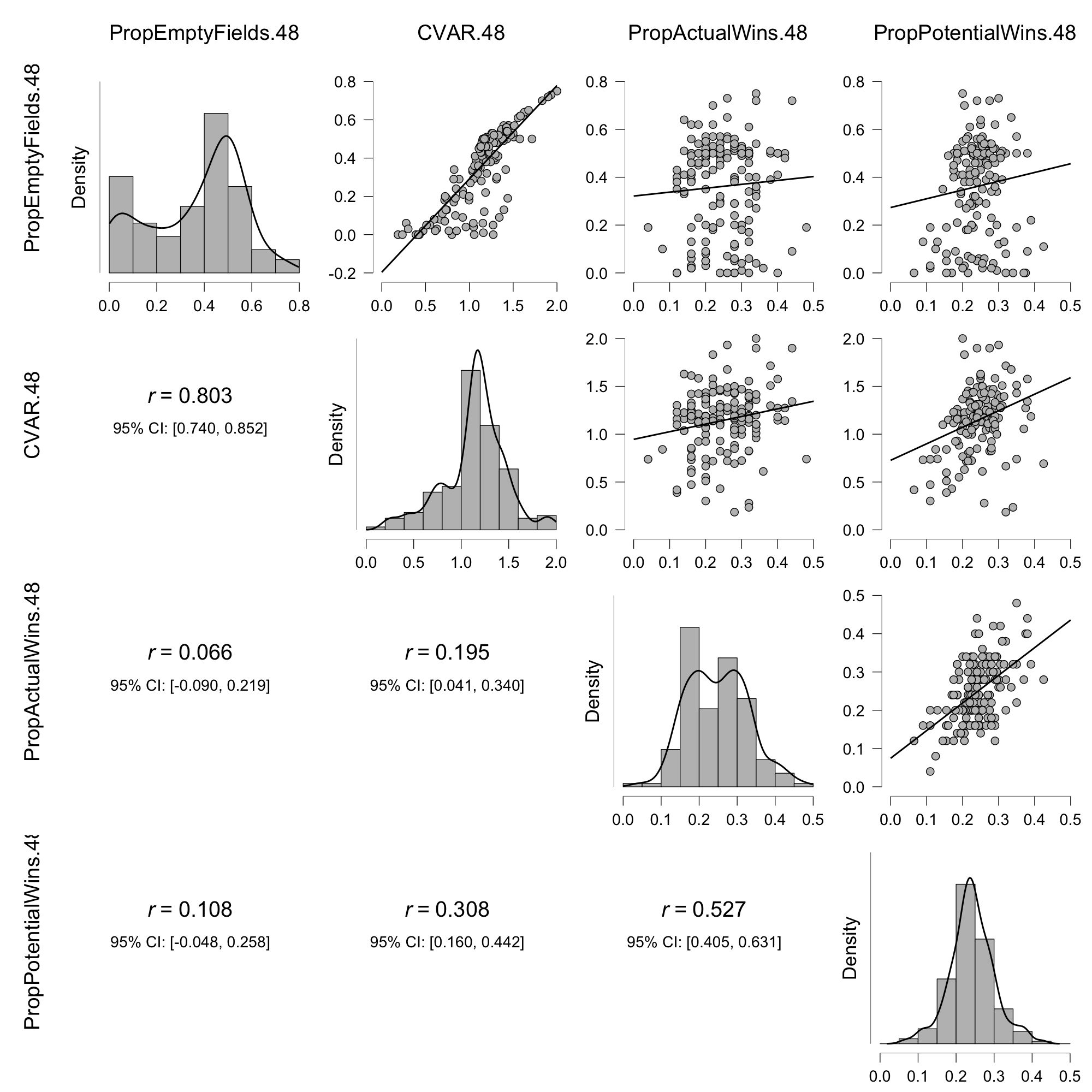


**Figure S8.** Asymmetric competition: Weaker players (48 vs. 96 points).


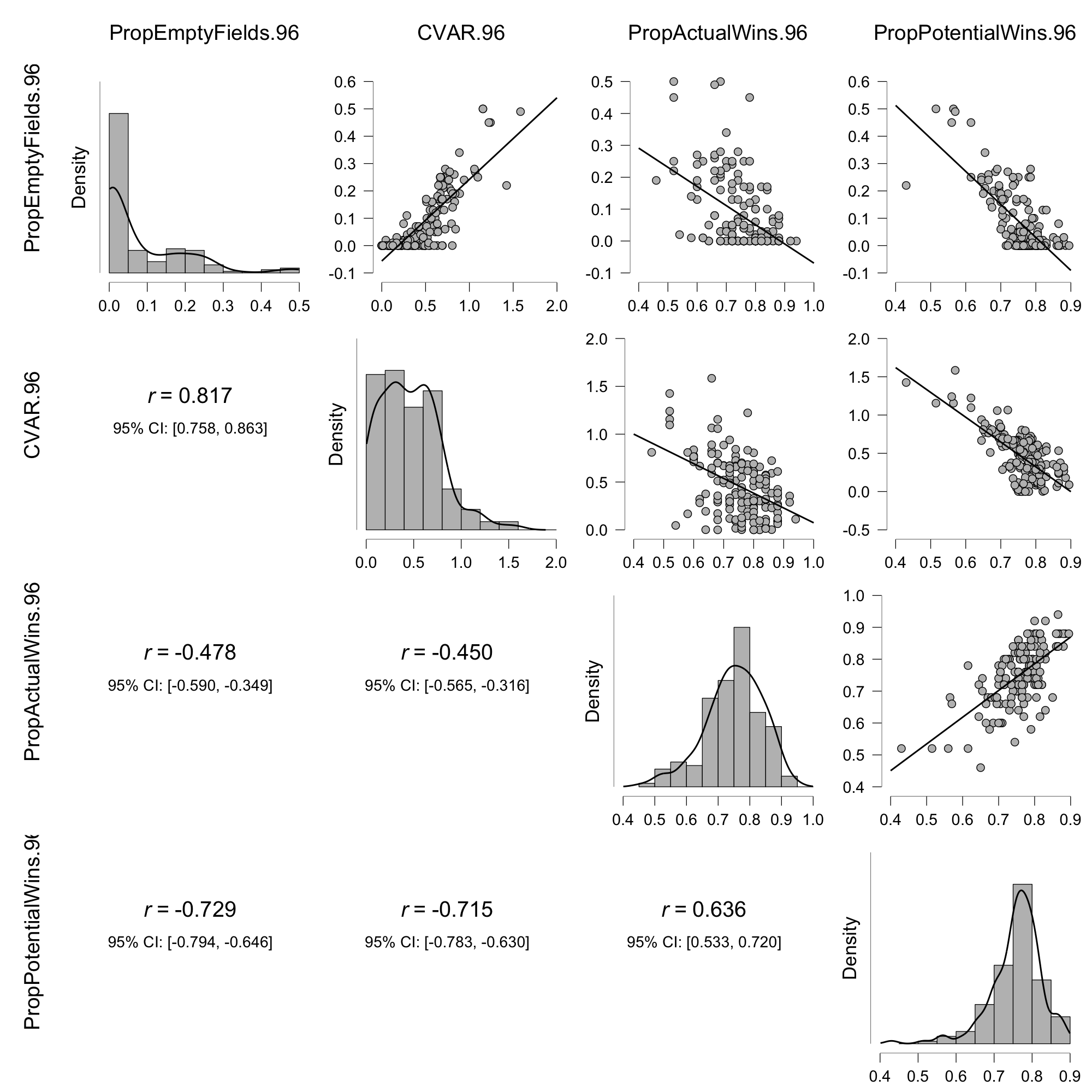


**Figure S9.** Asymmetric competition: Stronger players (96 vs. 48 points).


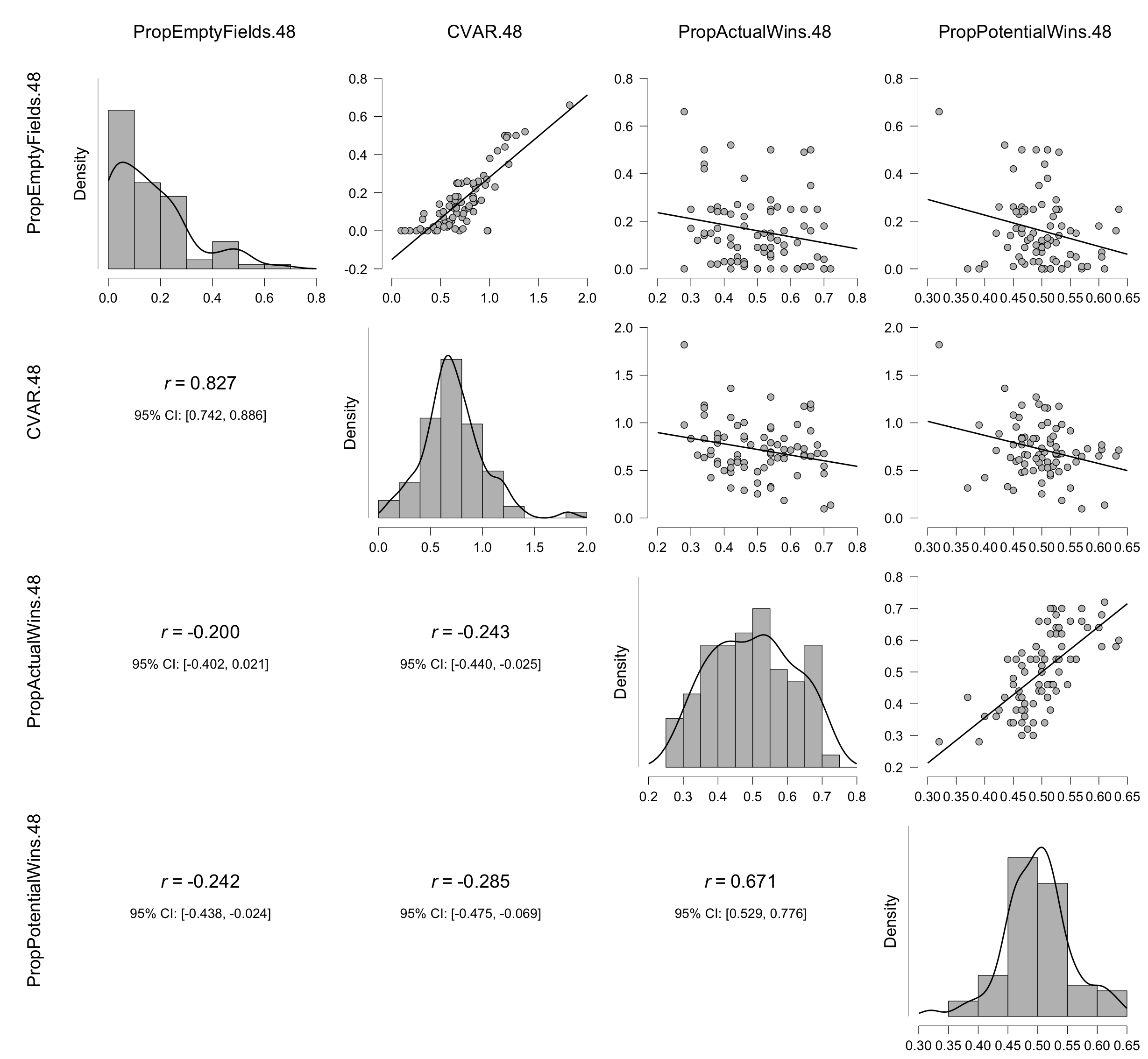


**Figure S10.** Symmetric competition: Equally strong players (48 vs. 48 points).


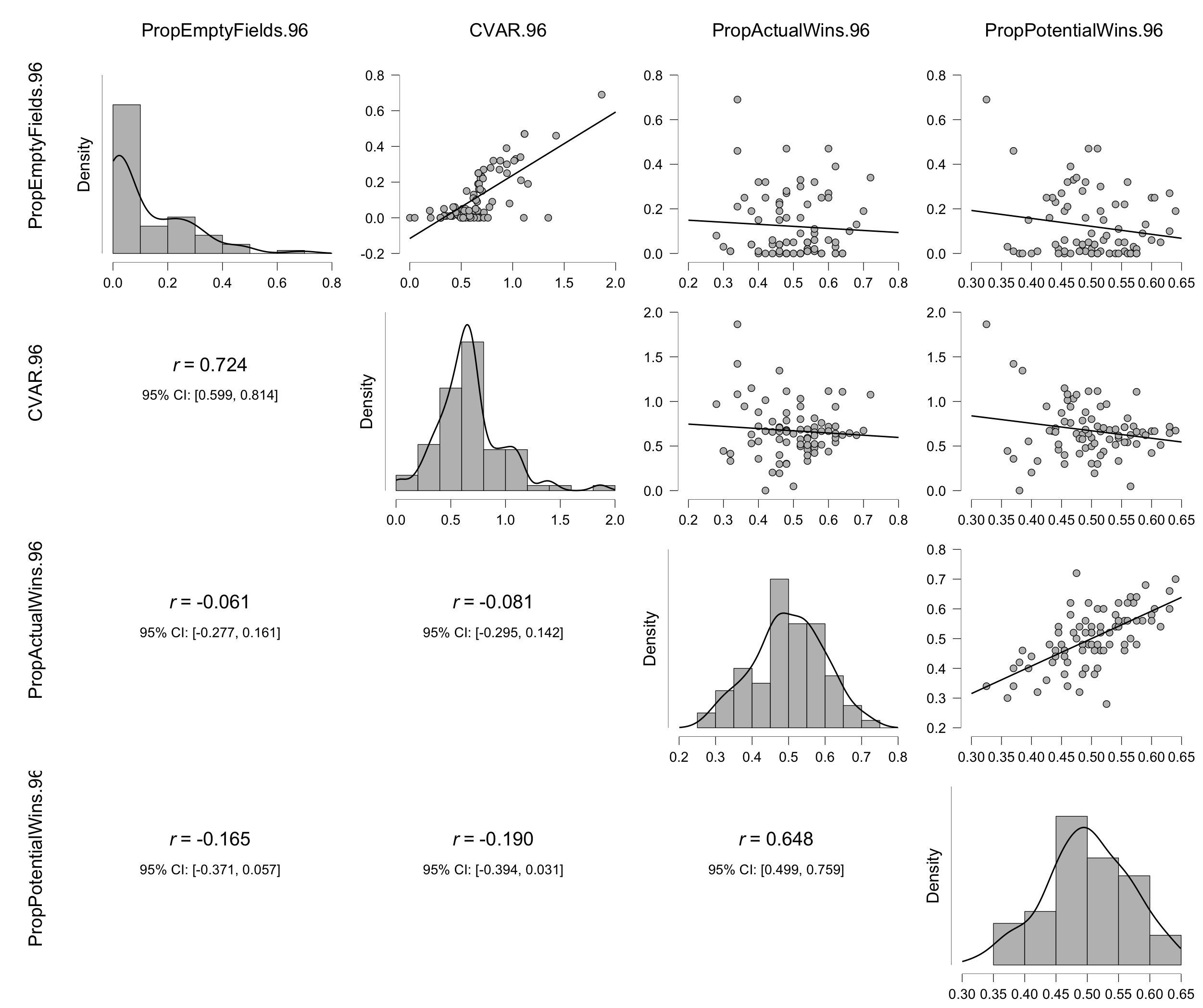


**Figure S11.** Symmetric competition: Equally strong players (96 vs. 96 points).

**Supplement 7: Zero-Order Correlations Among Individual-Difference Measures**

Using psychometric tests, we collected the following individual-difference measures from each participant in Study 1 and 2: crystallized abilities (verbal knowledge), fluid cognitive abilities (cognitive speed and reasoning), numeracy, positive and negative affect before the game, risk preferences, and social value orientation. Assuming that the relations between basic cognitive processes and game behavior held across studies, we combined the data from both studies to obtain more reliable estimates between these measures from the correlational analyses (cf. ^11^). Regressions are thus based on individual-difference measures from *N* = 240 participants (*n* = 160 in asymmetric competition and *n* = 80 in symmetric competition). For the correlational analyses, all variables were *z*-scaled across all participants (*M* = 0; *SD* = 1).


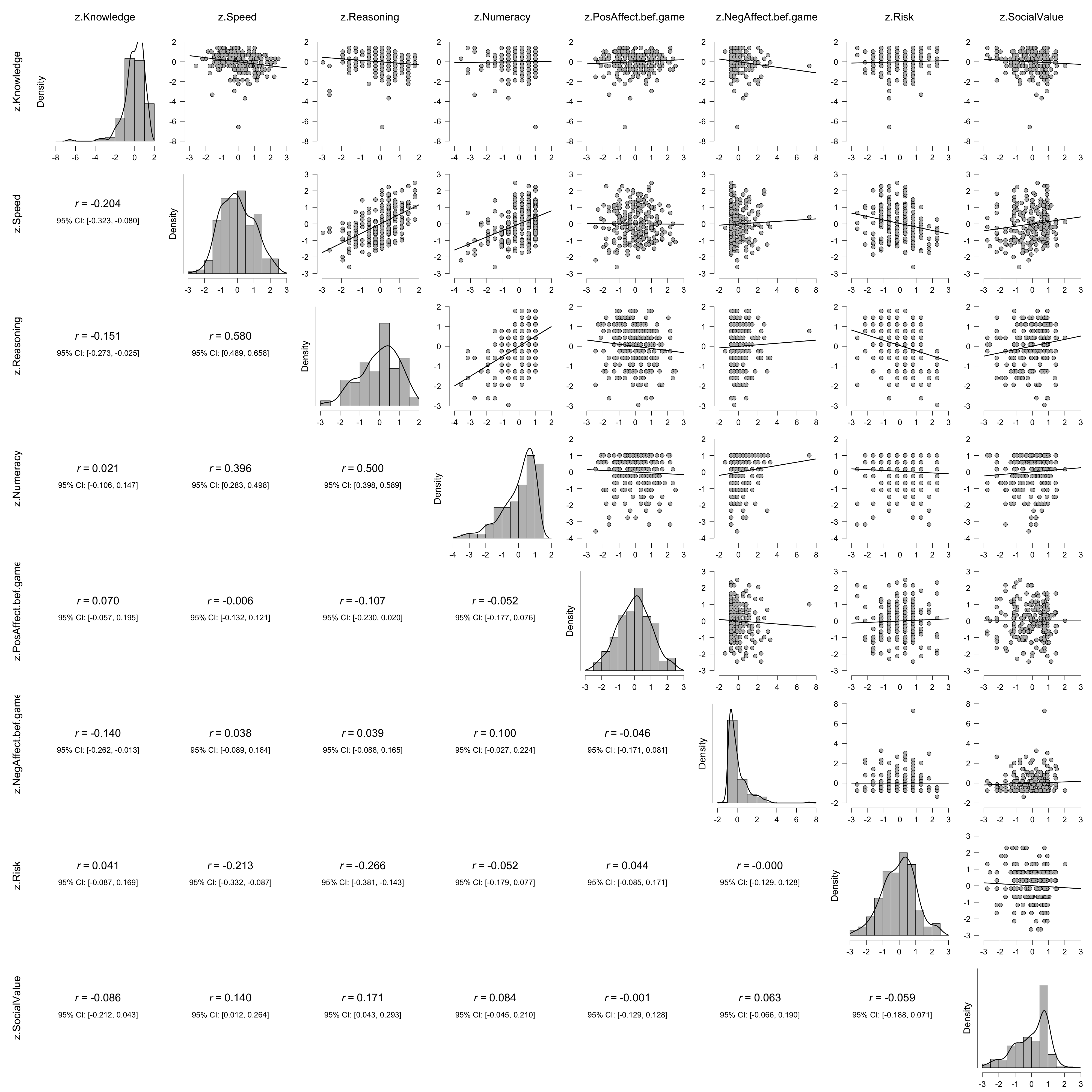


**Figure S12.** Zero-order correlations between psychometric test scores.

**Supplement 8: Analyses with Generalized Mixed-Models**

**Table S6.** *Mixed-Model Logistic Regressions of Allocation Behavior (Fields Left Empty) in Study 1.*

| Fixed effects | *M*_0_ | | *M*_1_ | | *M*_2_ | | *M*_3_ | |
| --- | --- | --- | --- | --- | --- | --- | --- | --- |
| Intercept (γ_00_) | 0.17*** | [0.13, 0.21] | 0.12*** | [0.09, 0.16] | 0.12*** | [0.09, 0.16] | 0.08*** | [0.06, 0.11] |
| Resources |  |  | 0.47*** | [0.45, 0.49] | 0.56*** | [0.53, 0.58] | 0.35*** | [0.28, 0.43] |
| AgeGroup |  |  | 0.72* | [0.56, 0.92] | 0.77* | [0.59, 1.00] | 0.77 | [0.56, 1.07] |
| OpponentStrength |  |  | 1.55** | [1.19, 2.03] | 1.34* | [1.03, 1.74] | 1.23 | [0.89, 1.71] |
| Resources×AgeGroup |  |  |  |  | 1.14*** | [1.10, 1.19] | 1.14 | [0.93, 1.39] |
| Resources×OpponentStrength |  |  |  |  | 0.66*** | [0.63, 0.69] | 0.65*** | [0.53, 0.79] |
| AgeGroup×OpponentStrength |  |  |  |  | 1.15 | [0.88, 1.50] | 1.21 | [0.87, 1.68] |
| Resources×AgeGroup×Opponent  Strength |  |  |  |  | 1.24*** | [1.19, 1.29] | 1.30** | [1.07, 1.58] |
| Random effects |  |  |  |  |  |  |  |  |
| Between-person intercept (τ_00_) | 1.95 |  | 1.89 |  | 1.85 |  | 2.77 |  |
| Slope variance (Strength) |  |  |  |  |  |  | 0.88 |  |
| Covariance |  |  |  |  |  |  | 0.63 |  |
| Further indices |  |  |  |  |  |  |  |  |
| Marginal *R*^2^ | 0 |  | 0.14 |  | 0.15 |  | 0.21 |  |
| ICC | 0.37 |  |  |  |  |  |  |  |
| BIC | 21427 |  | 19735 |  | 19192 |  | 18543 |  |
| log likelihood | −10703 |  | −9842 |  | −9551 |  | −9216 |  |

*Note.* The fixed effects are given as odds ratios exp (*B*) with 95% confidence intervals in brackets. The four model variants (*M*_0_ to *M*_3_) differ in included main, interaction, and random effects. The analysis with model *M*_3_ (with lowest the BIC) led to similar conclusions as reported in the main part of the manuscript. The random effects are reported as variances. Marginal *R*^2^ indicates the proportion of variance explained by the fixed effects. In the logistic regression of allocation behavior (fields left empty), the dependent variable was equal to 1 if a participant decided to leave a given field empty in a given round of the game and 0 otherwise: *P*(empty field = 1) / *P*(empty field = 0). The variables AgeGroup (younger vs. older), Opponent Strength (equal vs. unequal), Resources (48 vs. 96 points) were entered as effect-coded predictors. **p* < .05; ***p* < .01; ****p* < .001.

**Table S7.** *Mixed-Model Logistic Regressions of Allocation Behavior (Fields Left Empty) in Study 2.*

| Fixed effects | *M*_0_ | | *M*_1_ | | *M*_2_ | | *M*_3_ | |
| --- | --- | --- | --- | --- | --- | --- | --- | --- |
| Intercept (γ_00_) | 0.15*** | [0.12, 0.19] | 0.10*** | [0.08, 0.13] | 0.10*** | [0.08, 0.13] | 0.08*** | [0.06, 0.10] |
| Resources |  |  | 0.43*** | [0.42, 0.45] | 0.50*** | [0.48, 0.52] | 0.40*** | [0.32, 0.49] |
| AgeGroup |  |  | 1.08 | [0.86, 1.35] | 1.23 | [0.97, 1.58] | 1.27 | [0.97, 1.67] |
| OpponentStrength |  |  | 1.62*** | [1.27, 2.08] | 1.31* | [1.03, 1.68] | 1.16 | [0.88, 1.52] |
| Resources×AgeGroup |  |  |  |  | 1.20*** | [1.15, 1.26] | 1.20 | [0.99, 1.46] |
| Resources×OpponentStrength |  |  |  |  | 0.63*** | [0.60, 0.66] | 0.54*** | [0.44, 0.66] |
| AgeGroup×OpponentStrength |  |  |  |  | 1.02 | [0.80, 1.31] | 1.04 | [0.80, 1.37] |
| Resources×AgeGroup×Opponent  Strength |  |  |  |  | 1.19*** | [1.14, 1.25] | 1.28* | [1.05, 1.55] |
| Random effects |  |  |  |  |  |  |  |  |
| Between-person intercept (τ_00_) | 1.57 |  | 1.55 |  | 1.55 |  | 1.85 |  |
| Slope variance (Strength) |  |  |  |  |  |  | 0.90 |  |
| Covariance |  |  |  |  |  |  | 0.31 |  |
| Further indices |  |  |  |  |  |  |  |  |
| Marginal *R*^2^ | 0 |  | 0.16 |  | 0.19 |  | 0.23 |  |
| ICC | 0.32 |  |  |  |  |  |  |  |
| BIC | 20717 |  | 18802 |  | 18304 |  | 17636 |  |
| log likelihood | −10348 |  | −9376 |  | −9107 |  | −8762 |  |

*Note.* The fixed effects are given as odds ratios exp (*B*) with 95% confidence intervals in brackets. The four model variants (*M*_0_ to *M*_3_) differ in included main, interaction, and random effects. The analysis with model *M*_3_ (with lowest the BIC) led to similar conclusions as reported in the main part of the manuscript. The random effects are reported as variances. Marginal *R*^2^ indicates the proportion of variance explained by the fixed effects. In the logistic regression of allocation behavior (fields left empty), the dependent variable was equal to 1 if a participant decided to leave a given field empty in a given round of the game and 0 otherwise: *P*(empty field = 1) / *P*(empty field = 0). The variables AgeGroup (younger vs. older), Opponent Strength (equal vs. unequal), Resources (48 vs. 96 points) were entered as effect-coded predictors. **p* < .05; ***p* < .01; ****p* < .001.

**Table S8.** *Mixed-Model Logistic Regressions of Allocation Behavior with Opponent Age as Additional Effect (Study 1 vs. 2).*

|  | *M*_0_ | | *M*_1_ | | *M*_2_ | | *M*_3_ | |
| --- | --- | --- | --- | --- | --- | --- | --- | --- |
| Fixed effects |  |  |  |  |  |  |  |  |
| Intercept (γ_00_) | 0.16*** | [0.13, 0.19] | 0.11*** | [0.09, 0.13] | 0.11*** | [0.09, 0.14] | 0.08*** | [0.06, 0.10] |
| Resources |  |  | 0.45*** | [0.44, 0.47] | 0.53*** | [0.51, 0.54] | 0.37*** | [0.32, 0.43] |
| AgeGroup |  |  | 0.88 | [0.74, 1.04] | 0.97 | [0.81, 1.17] | 1.00 | [0.81, 1.23] |
| OpponentStrength |  |  | 1.59*** | [1.32, 1.91] | 1.33** | [1.11, 1.59] | 1.19 | [0.96, 1.46] |
| OpponentAge |  |  | 0.88 | [0.63, 1.24] | 0.83 | [0.58, 1.19] | 0.86 | [0.57, 1.31] |
| Resources×AgeGroup |  |  |  |  | 1.17*** | [1.14, 1.21] | 1.18* | [1.03, 1.36] |
| Resources×OpponentStrength |  |  |  |  | 0.64*** | [0.62, 0.66] | 0.59*** | [0.51, 0.68] |
| Resources×OpponentAge |  |  |  |  | 0.90*** | [0.84, 0.96] | 1.04 | [0.79, 1.36] |
| AgeGroup×OpponentStrength |  |  |  |  | 1.09 | [0.91, 1.30] | 1.13 | [0.91, 1.39] |
| AgeGroup×OpponentAge |  |  |  |  | 1.60* | [1.12, 2.30] | 1.62* | [1.07, 2.46] |
| OpponentStrength×OpponentAge |  |  |  |  | 0.99 | [0.69, 1.42] | 0.94 | [0.62, 1.43] |
| Resources×AgeGroup×OpponentStrength |  |  |  |  | 1.21*** | [1.18, 1.25] | 1.29*** | [1.12, 1.48] |
| Resources×AgeGroup×OpponentAge |  |  |  |  | 1.05 | [0.99, 1.12] | 1.03 | [0.79, 1.36] |
| Resources×OpponentStrength×OpponentAge |  |  |  |  | 0.95 | [0.89, 1.01] | 0.84 | [0.64, 1.10] |
| AgeGroup×OpponentStrength×OpponentAge |  |  |  |  | 0.89 | [0.62, 1.27] | 0.87 | [0.57, 1.32] |
| Resources×AgeGroup×OpponentStrength×OpponentAge |  |  |  |  | 0.96 | [0.91, 1.03] | 0.99 | [0.75, 1.30] |
| Random effects |  |  |  |  |  |  |  |  |
| Between-person intercept (τ_00_) | 1.77 |  | 1.76 |  | 1.70 |  | 2.26 |  |
| Slope variance (Strength) |  |  |  |  |  |  | 0.88 |  |
| Covariance |  |  |  |  |  |  | 0.47 |  |
| Model indices |  |  |  |  |  |  |  |  |
| BIC | 42127 |  | 38516 |  | 37499 |  | 36169 |  |

*Note.* The fixed effects are given as odds ratios exp (*B*) with 95% confidence intervals in brackets. The four model variants (*M*_0_ to *M*_3_) differ in included main, interaction, and random effects. The analysis with model *M*_3_ (with lowest the BIC) led to similar conclusions as reported in the main part of the manuscript. The random effects are reported as variances. In the logistic regression of allocation behavior (fields left empty), the dependent variable was equal to 1 if a participant decided to leave a given field empty in a given round of the game and 0 otherwise: *P*(empty field = 1) / *P*(empty field = 0). The variables AgeGroup (younger vs. older), Opponent Strength (equal vs. unequal), Resources (48 vs. 96 points) were entered as effect-coded predictors. **p* < .05; ***p* < .01; ****p* < .001.

**Supplement 9: Verbatim Screen Instructions for the Colonel Blotto Game**

This section provides the verbatim instructions [translated from German] that participants read for the Colonel Blotto game along with sample screenshots from the game. In addition, a trained experimenter explained the rules in standardized form and answered open questions before the game started.

1. Welcome
   - *“Welcome to our study. First, you will receive some information about the procedures. Please read all instructions carefully. After you have read a text page, please press the spacebar to move on to the next page... To move back to a previous page, press the left arrow key.”*
   - *“Please do not talk to others present during the study. If you have any questions later, please quietly raise your hand or go to the experimenter. Press the spacebar to move on.”*
   - *“Do you normally need a visual aid for computer screens (glasses/contact lenses)? Then please use them now. Please switch off your mobile phone if necessary. Press the spacebar to move on.”*
2. Information about the procedure
   - *“In this study, you play via the computer against other people who are present in the room now. The game has several rounds. In each round, you will compete against one of the people present (your opponent). The computer program determines which person you play against in a given round. Press the spacebar to move on.”*
   - *“In each round, the aim is to cleverly distribute resources (quantities of points) available to you across various fields. Your opponent in each round does the same. A round is completed once both players have made their allocation decisions. Press the spacebar to move on.”*
   - *“In the following, we will show you how the distribution game works on the computer. You will next see some ‘screen shots’ of the game as examples... Press the spacebar to move on.”*
3. The Allocation Game
   - *“The amount of resources available to you in each round is indicated by a gold-filled ‘jar’ (bin) on the left side of the screen.”*

**

**

**

**Figure S13.** Introductory screenshots for the competition game.

1. The goal of the game
   - *“The computer selects one of the four fields randomly (with equal probability). You will compete against your opponent on this one field. It now depends which player has allocated more resources to this field.”*
   - *“The player with more resources on this field wins the round. The goal of each round is to win against your opponent. If you win, you gain points.”*
2. Rewards
   - *“If you win a round, you get 2 points. If you lose a round, you get 0 points. In the case of a tie, the points are split. The points will be converted into euros at the end of the study. You can earn up to 10 euros! Press the spacebar to move on.”*
3. Example
   - *“Here is an example: After the players distributed their resources, the computer randomly selected the leftmost field. This field is marked with a red circle.”*

**

- - *“Player 1 gets a message that they have won 2 points.”*

**

- - *“In contrast, Player 2 gets a message that they have won 0 points.”*

**

- - *“Player 1 wins this example round because they put more resources on the first (leftmost) field than Player 2 did (25 vs. 24 units).”*

**

1. Instructions for the Asymmetric Condition
   - *“Important: In the following rounds, players competing against each other will differ in ‘strength’ (resources available). One player has 96 units in each round. The other player has 48 units in each round. Press the spacebar to move on.”*
   - *“So, if you have 96 units, this means that your opponent has 48 units (and vice versa!). After a first phase of 25 rounds and a short break, this relative strength will change...”*
   - *“In the second phase, the relative strength of the players will be reversed. The experimenter will let you know later in the break when the relative strengths change.”*
2. Instructions for the Symmetric Condition
   - *“Important: In the following rounds, players competing against each other will be equally ‘strong’ and have the same numbers of units at their disposal. Both players have 96 units in one phase. Both players have 48 units in another phase. Press the spacebar to move on.”*
   - *“After the first phase of 25 rounds and a short break, the numbers will change. The experimenter will let you know later during the break when the numbers change. But you will always play against equally strong opponents...”*
3. Final Remarks
   - *“The game will begin as soon as all participants are ready. To begin, you will play a short practice session to familiarize yourself with the task. Afterwards, you will play against the other participants for money. Press the spacebar to move on.”*
   - *“Now quietly raise your hand. Do you have any questions? Then please ask the experimenter.”*
4. Additional Instructions for Study 2 (Opponents from Different Age Groups)
   - *“In each round, you will compete against someone in this room who is significantly older or younger than you. So older adults will always play against younger adults! Who exactly you play against in a round is determined randomly.”*

**References to the Supplemental Online Materials**

1. Lehrl, S., Merz, J., Burkard, G. & Fischer, B. *Mehrfach-Wortschatz-Intelligenztest [Multiple Choice Vocabulary Intelligence Test]* (Perimed, 1991).
2. Lindenberger, U., Mayr, U. & Kliegl, R. Speed and intelligence in old age. *Psychol. Aging* **8**, 207–220 (1993).
3. Petermann, F. *Wechsler Adult Intelligence Scale WAIS-IV [German version of the Wechsler Adult Intelligence Scale]* (Pearson, 2012).
4. Weiß, R. H.. *CFT 20-R: Grundintelligenztest Skala [Culture Fair Intelligence Test 20]* (Hogrefe, 2006).
5. Lipkus, I. M., Samsa, G. & Rimer, B. K. General performance on a Numeracy Scale among highly educated samples. *Med. Decis. Mak.* **21**, 37–44 (2001).
6. Watson, D., Clark, L. A. & Tellegen, A. Development and validation of brief measures of positive and negative affect: The PANAS Scales. *J. Pers. Soc. Psych.* **54**, 1063–1070 (1988).
7. Holt, C. A. & Laury, S. K. Risk aversion and incentive effects. *Amer. Econ. Rev.* **92**, 1644–1655 (2002).
8. Murphy, R., Ackerman, K. & Handgraaf, M. Measuring social value orientation. *Judgm. Decis. Mak.* **6**, 771–781 (2011).
9. Avrahami, J. & Kareev, Y. Do the weak stand a chance? Distribution of resources in a competitive environment. *Cogn. Sci.* **33**, 940–950 (2009).
10. Avrahami, J., Kareev, Y., Todd, P. M. & Silverman, B. Allocation of resources in asymmetric competitions: How do the weak maintain a chance of winning? *J. Econ. Psychol.* **42**, 161–174 (2014).
11. Schönbrodt, F. D. & Perugini, M. At what sample size do correlations stabilize? *J. Res. Pers.* **47**, 609–612 (2013).
